# Coinfection and cross-reaction of dengue and COVID-19: a case series analysis

**DOI:** 10.1590/0037-8682-0243-2022

**Published:** 2022-10-21

**Authors:** Maria Emilia Avelar Machado, Elza Kimura

**Affiliations:** 1 Universidade Estadual de Maringá, Departamento de Medicina, Programa de Mestrado Profissional em Gestão, Tecnologia e Inovação em Urgência e Emergência, Maringá, PR, Brasil.; 2 Universidade Estadual de Maringá, Departamento de Medicina, Maringá, PR, Brasil.; 3 Universidade Estadual de Maringá, Departamento de Farmácia, Maringá, PR, Brasil.

**Keywords:** Dengue, COVID-19, Coinfection, Cross-reaction

## Abstract

**Background:**

The risk of possible cross-reactions between serological tests, together with the clinical similarities between dengue fever and COVID-19, can delay diagnosis and increase the risk of both COVID-19 transmission and worsening. The present study aimed to determine the possibility of cross-reactions among rapid serological tests based on clinical symptoms.

**Methods:**

Patients with COVID-19, confirmed by RT-PCR and clinical criteria for diagnosing dengue, were recruited consecutively between September 2020 and August 2021 and underwent rapid immunochromatographic diagnostic (RID) tests for AgNS1, IgM, and IgG. Patients who tested positive for acute-phase dengue IgM and AgNS1 underwent a follow-up test after 12-30 days for diagnostic confirmation.

**Results:**

A total of 43 patients were included, 38 of whom required hospital admission, and 8 received intensive care. Seven patients tested positive on the RID tests, comprising 2 NS1 positive (coinfection), one reactive for IgM and IgG (coinfection), three reactive for IgM not confirmed (false-positive), and one reactive for IgG due to previous infection. Two of the 3 patients with coinfection died. Fever, myalgia, headache, and cough were the most common clinical symptoms, while lymphopenia was the most prevalent laboratory finding.

**Conclusions:**

Cross-reactivity was found in only three patients and coinfection in another three patients, two of whom died of severe COVID-19 manifestations.

## INTRODUCTION

Dengue and coronavirus disease (COVID-19) are major public health problems in Brazil and are acute febrile diseases with a variety of similar clinical presentations that can progress to clinically severe forms^1^ ^-^ ^3^.

Rapid immunochromatographic diagnostic (RID) tests are important tools for early diagnosis of dengue and allow more adequate management of patients with acute febrile syndrome^1^. Despite the high specificity and sensitivity of the rapid test for dengue virus (DENV)^4^, different countries have reported cross-reactivity of dengue antibodies using RID tests among COVID-19 patients^5^ ^,^ ^6^.

The description of two cases of COVID-19 misdiagnosed as dengue in Singapore^7^ and a suspected case of dengue in Thailand with COVID-19 transmission^8^ sounded an alert regarding the possibility of serological cross-reactivity between the two viruses and its consequences. This led to a study carried out by an Israeli research group that found a false-positive reaction rate of approximately 22% between dengue and COVID-19, based on the results of IgM/IgG RID tests^9^. This cross-reaction may be explained by the similarity between the HR2 domain of the SAR-CoV2 spike protein and the E protein of DENV identified in an in-silico study^9^.

Many other countries have also reported cases of dengue and COVID-19 coinfection, some of which showed a worse evolution^10^ ^,^ ^11^. Dengue is diagnosed based on clinical, epidemiological, and laboratory data. RID tests provide determinations of NS1 (viral antigen) and IgM and IgG antibodies and are widely used by emergency services for the diagnosis of dengue. The sensitivity of these tests depends on the time since symptom onset^1^.

In diagnosing COVID-19, besides clinical and radiological evidence, nasal/oral swab samples are used for reverse transcription polymerase chain reaction (RT-PCR) or antigen tests^3^. RT-PCR offers high sensitivity and specificity, but yields results in approximately 24 h^3^ ^,^ ^12^. Antigen tests tend to yield faster results but provide lower sensitivity than RT-PCR^3^ ^,^ ^12^.

The possibility of cross-reaction of infection by the novel coronavirus with serological tests for dengue is a concern in countries where the two viruses coexist^5^ ^,^ ^13^ because, in addition to the possible impact on patient health, there is a risk of delay in adopting the measures needed to prevent transmission of severe acute respiratory syndrome caused by coronavirus 2 (SARS-CoV-2). In view of this risk, the objective of the present study was to follow up on patients presenting to the hospital with RT-PCR-confirmed COVID-19 and symptoms resembling dengue and to investigate the possibility of coinfection and cross-reactivity with dengue RID tests.

## METHODS

### Study type, population, and setting

A prospective observational case series study was conducted to assess the signs and symptoms suggestive of dengue in patients with confirmed COVID-19 and to determine the specificity of acute-phase serological tests for dengue IgM and NS1 in patients with confirmed COVID-19.

The study protocol was approved by the ethics review boards of two private hospitals in the city of Maringá (Paraná State) and by the Research Ethics Committee of the State University of Maringá under CAAE no. 36501620.5.0000.0104, permit no. 4.226.639, on the 20th of August 2020. The study population included patients treated at the emergency department and/or admitted to adult intensive care units (ICUs) and/or adult infirmary wards diagnosed with RT-PCR-confirmed COVID-19 using nasal/oral swab samples during the period from September 2020 to August 2021. Patients were selected by convenience sampling based on the signs and symptoms presented. The physician responsible for the admission of the patient was informed, and there was no influence on the therapeutic management of the patient. 

The inclusion criteria were patients older than 18 years, admitted with a diagnosis of COVID-19 confirmed via RT-PCR assay, in association with diagnostic clinical criteria for dengue (fever for up to 7 days and at least two of the following signs and symptoms: headache/retro-orbital pain, nausea/vomiting, myalgia, arthralgia, exanthem, petechiae, or leukopenia). The exclusion criteria were individuals diagnosed with COVID-19 via serology and/or clinical/epidemiological criteria with a history of dengue vaccination or prior dengue infection in the preceding 6 months.

### Procedure

Cases of COVID-19 were confirmed via real-time RT-PCR tests performed at laboratories approved by the National Ministry of Health. Serum samples were collected for rapid diagnostic (RID) testing using the OnSite Dengue IgM/IgG (CTK Biotech, San Diego, CA, USA) and OnSite Ag kits, and for detection of NS1 (Antigen CTK Biotech, San Diego, CA, USA). Lateral flow immunochromatography was carried out at the Núcleo Diagnóstico laboratory.

Patients who tested positive on the acute-phase dengue test (AgNS1 or IgM) underwent follow-up serological collection 12-30 days after the first test. Data on anthropometric and clinical characteristics of the patients were collected and registered in charts (containing the following information: date of admission, date of symptom onset, date of hospital discharge, sex, age, race, presence of comorbidities, prior dengue infection, and dengue vaccination history. Patients were probed for the presence, on admission or prior to admission, of the following clinical variables: clinically measured or reported fever, myalgia, arthralgia, headache, diarrhea, nausea, vomiting, abdominal pain, exanthem, cough, expectoration, coryza, nasal congestion, sore throat, respiratory distress, chest pain, and shortness of breath. 

The following data were collected from laboratory examinations on the admission day: hematocrit, hemoglobin level, total leukocyte count, total lymphocyte count, platelets, oxygen saturation assessed via percutaneous (pulse) oximetry in ambient air, aspartate aminotransferase level, alanine aminotransferase level, and C-reactive protein (CRP) level. The secondary outcomes were the need for ICU admission, length of ICU stay, progression to mechanical ventilation, and duration of mechanical ventilation. 

### Statistical analysis

Descriptive analyses of anthropometric, clinical, and laboratory data were performed using the free software, JASP® Team Computer software 2021(version 0.14.1.0)^14^. Symptom onset, tracking of outcomes, length of ICU stay, and mechanical ventilation time were compared between patients with symptoms suggestive of dengue and COVID-19 coinfection. 

## RESULTS

### Patient characteristics and disease progression

A total of 43 patients that met the clinical criteria for suspected dengue and had RT-PCR-confirmed COVID-19 were included in the study. Of these, 38 required hospital admission, and only 5 patients were assessed in the emergency room. 

The patients had a mean age of 50.6 years, were predominantly male (60.5%), had a mean body mass index of 28.7 kg/m^2^, and 55.8% presented with comorbidities (Table 1). Arterial hypertension was the most prevalent comorbidity (25.5%), and 14% had two or more comorbidities. The mean duration of symptoms prior to admission was 6.4 days, with 8 patients requiring ICU admission and 6 requiring mechanical ventilation. Of the 43 patients included in the study, 5 died. 

**TABLE 1:** Anthropometric and clinical characteristics on admission.

Characteristics	Total	Living	Deaths
	n=43 (100%)	n=38 (88.4%)	n=5 (11.6%)
Male, n (%)	26 (60.5)	23 (60.5)	3 (60)
Race, white; n (%)	41 (95.3)	36 (94.7)	5 (100)
Age, years [mean (SD)] (range)	50.6 (12.2) (27-75)	48.5 (12.1) (27-75)	59.2 (9.3) (45-68)
BMI, kg/m^2^ [mean (SD)] (range)	28.7 (5.4) (18.6-42.9)	28.3 (5.5) (18.6-42.9)	31.4 (3.9) (26.8-36.8)
*Comorbidities*	24 (55.8)	19 (50)	5 (100)
Diabetes, n (%)	3 (7)	3 (7.9)	0
Hypertension, n (%)	11 (25.5)	7 (18.4)	4 (80)
Other, n (%)	17 (39.5)	16 (42.1)	1 (20)
Two or more, n (%)	6 (14)	6 (15.8)	0
Symptom duration pre-admission, days [mean (SD)] (range)	6.4 (3.0) (2-15)	6.5 (3.1) (2-15)	6.2 (2.2) (3-9)
**Laboratory data**			
Leukopenia, n (%)	12 (27.9)	10 (26.3)	2 (40)
Lymphocytopenia, n (%)	29 (67.4)	24 (63.2)	5 (100)
Thrombocytopenia, n (%)	10 (23.3)	9 (23.7)	1 (20)
Oxygen saturation <94%, n (%)	9 (21.4)	8 (21.6)	1 (20)
**Hospital data**			
Inpatients, n	n=38	n=33	n=5
Length of stay, days [mean (SD)] (range)	16.2 (20.6) (3-92)	11.8 (14.5) (3-91)	44.6 (35.0) (7-92)
ICU admission, n (%)	8 (21.1)	4 (12.1)	4 (80)
ICU stay, days [mean (SD)] (range)	36.4 (19.4) (4-90)	23.3 (10.8) (4-64)	49.5 (37.1) (6-90)
MV, n (%)	6 (15.8)	2 (6.06)	4 (80)
Time on MV, days [mean (SD)] (range)	41.2 (17.1) (6-80)	31 (7.9) (15-47)	46.3 (33.5) (6-80)

**SD:** standard deviation; **BMI:** body mass index; **ICU:** intensive care unit; **MV:** mechanical ventilation.

**FIGURE 1:**
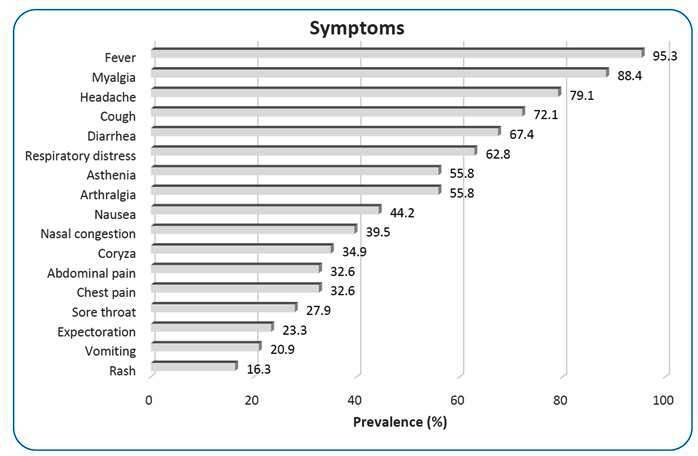
Main symptoms presented on admission. The symptoms reported on admission are expressed as percentage occurrence.

With regard to laboratory biomarkers, a majority of patients had lymphopenia (67.4%), 27.9% had leukopenia, and 23.3% had thrombocytopenia. For a prior history of dengue, 11 patients (25.58%) reported having dengue more than 1 year prior, and only two (4.7%) patients reported previous vaccination for dengue. The most frequently reported symptoms on admission were fever (95.3%), myalgia (88.4%), and headaches (79.1%). Other symptoms presented by the patients on admission included cough (72.1%), respiratory distress (62.8%), nasal congestion (39.5%), coryza (34.9%), and sore throat (27.9%) (Figure 1). 

### Diagnostic tests for dengue

Of the patients included, seven (16.3%) tested positive on at least one diagnostic test for dengue on admission. Among these patients, two tested positive on the NS1 antigen test, three on the IgM test, one on the IgG test, and one was reactive for both IgM and IgG.

Patients who tested positive on serological tests were investigated for the possibility of coinfection or cross-reaction (false-positive) according to the results of the initial and follow-up RID tests (Table 2). Clinical symptoms were similar between coinfected patients and those with positive cross-reactions. All patients had elevated CRP concentrations (Table 3).

**TABLE 2:** Characteristics of patients who tested positive on serological dengue test.

Patient no.	Prior dengue infection	Symptom duration, days	Initial serology findings			Findings on serology ≥12 days			Conclusion
			NS1	IgM	IgG	NS1	IgM	IgG	
**37**	Yes	3	+	-	-	-	+	-	**Coinfection**
**36**	No	2	+	-	-	NP	NP	NP	Inconclusive, probable coinfection
**6**	Yes	7	-	+	-	-	+	+	**Coinfection**
**8**	Yes	10	-	+	+	-	-	-	False-positive
**30**	Yes	1	-	+	-	-	-	-	False-positive
**43**	No	3	-	+	-	NP	NP	NP	Inconclusive, probable false-positive
5	Yes	8	-	-	+	NP			Previous dengue

**NP:** not performed; **NS1:** viral NS1 antigen; **IgM:** immunoglobulin M; **IgG:** immunoglobulin G.

**TABLE 3:** Clinical and laboratory characteristics of patients with coinfection or false-positive results.

	Patient characteristics					
	Coinfection			Cross reaction		
	Patient 6	Patient 36	Patient 37	Patient 8	Patient 30	Patient 43
**General symptoms**	Fever, myalgia, arthralgia, headache, diarrhea, nausea, abdominal pain, and asthenia	Fever, arthralgia, myalgia, headache, and diarrhea	Fever, headache, diarrhea, nausea, and vomiting	Fever, headache, diarrhea, nausea, and asthenia	Fever, myalgia, arthralgia, diarrhea, nausea, vomiting, abdominal pain, and asthenia	Fever, myalgia, arthralgia, headache, asthenia, diarrhea, and retro-ocular pain
**Respiratory symptoms**	Cough and coryza	Cough, sore throat, and respiratory distress	Cough, coryza, nasal congestion, sore throat, and respiratory distress	Cough	Cough, nasal congestion, and respiratory distress	Cough
**Evidence**	Prior dengue infection over 5 years. Tested IgM positive on day 7 of symptoms. On the 29^th^ day, the patient tested IgM and IgG positive for dengue.	No previous dengue. On the 2^nd^ day of symptoms, the patient tested NS1 positive. On the 10^th^ day, the patient tested IgG, IgM, and NS1 negative. Case not concluded due to death of the patient. Possible coinfection.	Prior dengue infection over 14 years. On the 3^rd^ day, the patient tested NS1 positive and IgM/IgG negative. On the 39^th^ day, the patient tested IgM positive and NS1 and IgG negative.	Prior dengue infection over 1 year. On the 10^th^ day, the patient tested IgM and IgG positive. On the 25^th^ day, the patient tested both IgM and IgG negative.	Prior dengue over 1 year. On the 1^st^ day, the patient tested NS1 negative and IgM positive. On the 12^th^ day, the patient tested NS1, IgM, and IgG negative.	No previous dengue. On the 3^rd^ day, the patient tested IgM positive and NS1 and IgG negative. The patient refused to repeat the test. Case not concluded, but likely false-positive.
**Outcome**	Critical case of COVID-19, requiring ICU admission and mechanical ventilation. The patient **died** on day 55 of the hospital stay.	Dengue was diagnosed on the 2^nd^ day of symptoms and COVID-19 on the 6^th^ day. Severe COVID-19. Presented with pulmonary embolism and **died** without ICU admission.	A moderate case of COVID-19 and **discharged** on the 11^th^ day of hospital stay.	A moderate case of COVID-19 and **discharged** on the 7^th^ day of hospital stay.	Critical case of COVID-19, requiring ICU admission and mechanical ventilation. The patient **died** on the 95^th^ day of hospital stay.	Moderate COVID-19 and **discharged** on 7^th^ day of hospital stay
**Leukocyte count <4000 /mm** ^3^	No	No	No	No	No	Yes
**Lymphocyte count <1000 /mm** ^3^	Yes	Yes	Yes	No	Yes	Yes
Platelet count <150,000 /mm^3^	No	No	Yes	No	No	Yes
CRP level	Elevated	Elevated	Elevated	Elevated	Elevated	Elevated

**CRP:** C-reactive protein; **NS1:** viral NS1 antigen; **IgM:** immunoglobulin M; **IgG:** immunoglobulin G; **ICU:** intensive care unit.

**Note:** Patient 5 was excluded from the analysis due to classification as a case of previous dengue infection.

In addition to the symptoms and alterations in laboratory parameters, other characteristics of the trajectory of patients who tested positive for dengue are outlined below, together with evidence for the classification of possible infection, cross-reaction, or previous infection, which are summarized in Table 3. Two of the 3 patients with possible coinfections died.

Patient 5 was not included in Table 3 because he had evidence of previous dengue infection based on RID tests for dengue, and tested positive for IgG and negative for IgM and NS1. He also had a history of dengue infection 2 years prior. He was admitted to the hospital on the 8^th^ day of symptoms, progressed to a moderate case of COVID-19, and was discharged on the 4^th^ day of his hospital stay.

## DISCUSSION

The difficulty in differentiating dengue from COVID-19 is a cause for concern in countries with a high incidence of both viruses^15^ ^-^ ^18^. The present study confirmed this diagnostic difficulty by presenting a case series of patients who met the criteria for suspected dengue and had a clinically confirmed diagnosis of COVID-19. 

**FIGURE 2:**
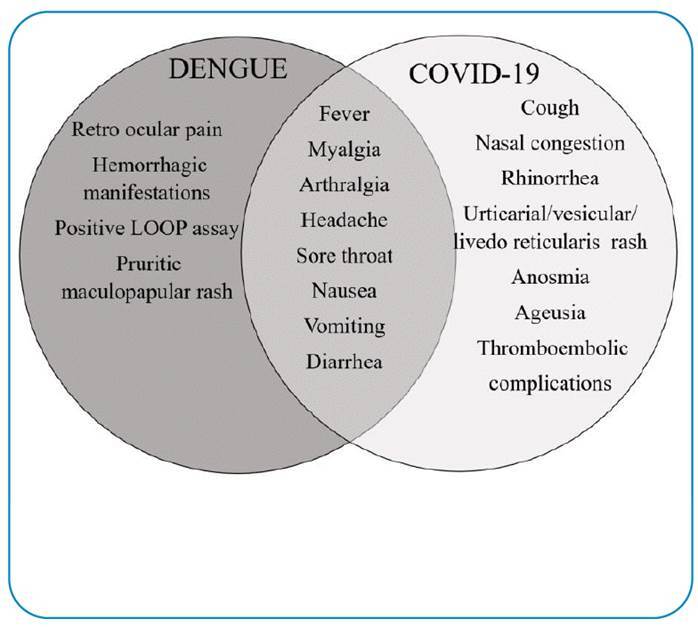
Main symptoms of Dengue or Covid-19 and overlapping symptoms. Main symptoms presented by the patients infected with dengue (dark gray) or Covid-19 (light gray) and the common symptoms presented by both diseases.

Although respiratory symptoms are important in cases of suspected COVID-19, unspecific systemic symptoms, such as fever, headache, and myalgia, were most common in the present study, followed by cough, diarrhea, and respiratory distress, due to the inclusion criteria of this investigation. Compared with data from the Centers for Disease Control and Prevention, the most common symptom among inpatients with COVID-19 was shortness of breath, while less than half of the patients presented with fever on hospital admission^19^. Among outpatients, however, fatigue, headache, and myalgia followed by sore throat, nasal congestion, and rhinorrhea were more frequent^19^. Thus, in outpatients with COVID-19, the predominance of systemic symptoms hampers the differential diagnosis. The symptoms common to both diseases, together with the symptoms that differentiate them, are shown in Figure 2.

Cough is a frequent respiratory symptom of COVID-19 but is uncommon in dengue^12^ ^,^ ^20^. Studies comparing COVID-19 and dengue confirmed that systemic symptoms, such as fever, myalgia, and headache, predominate over respiratory symptoms in dengue^17^ ^,^ ^21^.

In the present case series, only 27.8% of patients presented with leukopenia and 23.3% with thrombocytopenia, a characteristic of dengue, while lymphopenia, a common feature of COVID-19, was the most prevalent manifestation (67.4%). This result mirrors the studies conducted by Rosso et al. (2021), who found higher rates of leukopenia and thrombocytopenia in dengue patients^17^ and by Gupta et al. (2020), who also found a high prevalence of lymphopenia in COVID-19 patients^22^. In studies of cases with coinfection, fever was the most common symptom, followed by dyspnea, fatigue, and headache. The most prevalent laboratory findings were thrombocytopenia and lymphopenia^11^.

The two viruses show differences in clinical progression, which can be useful in their differential diagnoses. While in dengue, a sudden decrease in temperature between the 3^rd^ and 5^th^ days is a sign of severity^1^, in COVID-19, fever may persist for over 7 days, commence or worsen later in patients who progress to severe forms^15^, and may not be present at all in older adults^23^.

Several cases of coinfection have been reported in many countries^6^ ^,^ ^10^ ^,^ ^24^ ^-^ ^29^. However, studies of coinfection have yielded conflicting results. In a study of 13 cases of coinfection in Argentina, Carosella et al. (2021) found a predominance of systemic symptoms, fever in all patients, and no deaths^10^. In contrast, a study involving 50 cases of coinfection found fever in 52% of patients and a greater rate of progression to death, and most of these cases had alarm signs or severe dengue^29^. However, unlike the present study and the Argentinian investigation, the cited study did not confirm COVID-19 diagnosis using RT-PCR, possibly contributing to the diagnostic uncertainty. 

Coinfection with dengue as a factor for severity for patients with COVID-19 and its association with mortality have also been observed^11^. Moreover, prior dengue infection was found to confer no protection against COVID-19^30^ and contributed to a greater rate of hospitalization^31^. Although the present study involved only a small number of cases, two of the three coinfected patients died.

The occurrence of cross-reaction with the dengue antigen and antibody test among flaviviruses, such as Zika and yellow fever viruses^32^, has been described. However, cross-reactivity of serological IgM and IgG antibody tests in patients with COVID-19 and dengue has been observed both in the present study and by several other authors^6^ ^,^ ^9^ ^,^ ^33^. Lustin et al.^9^ found a 22% false-positivity rate using dengue IgM/IgG RID tests in patients with RT-PCR-diagnosed COVID-19 in a non-endemic region for dengue, although these results were not confirmed when repeated using the ELISA technique. In the present study, only three (7%) patients had possible cross-reactions, a pattern similar to that reported by other authors^34^ ^,^ ^35^. A study conducted in Indonesia that assessed 95 RT-PCR-confirmed COVID cases based on dengue RID tests detected only one IgM-positive case, one IgG-positive case, and one probable case of coinfection with positivity for NS1, IgM, and IgG^34^. In an Italian study, no IgM/IgG positivity for dengue was found in 32 patients diagnosed with COVID-19, although the tests were performed using ELISA^36^. In Colombia, a study on the accuracy of the ELISA IgM/IgG test for SARS-CoV-2 in different groups found no positivity in serum samples of patients diagnosed with dengue in 2019^37^. 

However, a study of 120 patients with confirmed COVID-19 in Singapore, also analyzing the accuracy of the dengue RID tests, found positivity for IgG in only four patients^38^ classified as having a previous infection. Unlike the present study, the patients’ symptoms were not assessed. Akin to the present investigation, none of the cited studies reported cross-reactions with NS1. 

The present study had some limitations. First, a small number of patients were included, explained by the inclusion criteria of symptoms of suspected dengue. Second, low circulation of DENV in the city during the study period may have contributed to the occurrence of a few cases of coinfection. Third, the method used for diagnosing dengue was the RID test, and DENV positivity was not confirmed via RT-PCR or ELISA. This shortcoming was partially remedied by repeating the RID tests after 12-40 days in patients who tested positive for acute-phase dengue (NS1 antigen and IgM antibody).

Similar to other studies, the present study identified both patients with positive cross-reactions and those with coinfection^6^ ^,^ ^33^. These findings highlight the need for screening tests that can offer greater diagnostic accuracy than RID tests, and for protocols that help physicians treat patients with acute febrile syndrome in dengue and COVID-19 endemic regions, particularly during a period outside the peak incidence of the two diseases. 
